# Injury Severity and Mortality of Adult Zebra Crosswalk and Non-Zebra Crosswalk Road Crossing Accidents: A Cross-Sectional Analysis

**DOI:** 10.1371/journal.pone.0090835

**Published:** 2014-03-03

**Authors:** Carmen A. Pfortmueller, Mariana Marti, Mirco Kunz, Gregor Lindner, Aristomenis K. Exadaktylos

**Affiliations:** 1 University Department of General Internal Medicine, University Hospital and University of Bern, Bern, Switzerland; 2 University Department of Emergency Medicine, University Hospital and University of Bern, Bern, Switzerland; 3 Department of Anesthesiology, Kantonsspital Winterthur, Winterthur, Switzerland; University of Florida College of Medicine, United States of America

## Abstract

**Principals:**

Over a million people worldwide die each year from road traffic injuries and more than 10 million sustain permanent disabilities. Many of these victims are pedestrians. The present retrospective study analyzes the severity and mortality of injuries suffered by adult pedestrians, depending on whether they used a zebra crosswalk.

**Methods:**

Our retrospective data analysis covered adult patients admitted to our emergency department (ED) between 1 January 2000 and 31 December 2012 after being hit by a vehicle while crossing the road as a pedestrian. Patients were identified by using a string term. Medical, police and ambulance records were reviewed for data extraction.

**Results:**

A total of 347 patients were eligible for study inclusion. Two hundred and three (203; 58.5%) patients were on a zebra crosswalk and 144 (41.5%) were not. The mean ISS (injury Severity Score) was 12.1 (SD 14.7, range 1-75). The vehicles were faster in non-zebra crosswalk accidents (47.7 km/n, versus 41.4 km/h, p<0.027). The mean ISS score was higher in patients with non-zebra crosswalk accidents; 14.4 (SD 16.5, range 1–75) versus 10.5 (SD13.14, range 1–75) (p<0.019). Zebra crosswalk accidents were associated with less risk of severe injury (OR 0.61, 95% CI 0.38–0.98, p<0.042). Accidents involving a truck were associated with increased risk of severe injury (OR 3.53, 95%CI 1.21–10.26, p<0.02).

**Conclusion:**

Accidents on zebra crosswalks are more common than those not on zebra crosswalks. The injury severity of non-zebra crosswalk accidents is significantly higher than in patients with zebra crosswalk accidents. Accidents involving large vehicles are associated with increased risk of severe injury. Further prospective studies are needed, with detailed assessment of motor vehicle types and speed.

## Introduction

Over a million people worldwide die each year in road traffic injuries and more than 10 million people sustain permanent disabilities [1], [2]. The World Health Organization has reported that, for people aged 3–35 years, road traffic accidents are now the leading cause of death and disablement [2]. The global economic burden of road traffic crashes is estimated at 500 billion US dollar per year [2]. According to the Swiss Accident Prevention Agency (BFU), 24,237/1,000,000 people were involved in a traffic accident in 2011 in Switzerland [3]. Of these, 2249 were pedestrians, and 75 of these pedestrians died [3]. It has been estimated that one third of these traffic accidents take place on a zebra crosswalk (also known as zebra crossings or pedestrian crossings) [3]. In Switzerland, zebra crosswalks are the only type of street crossing that exists and pedestrians are intended to use them to cross the road. Zebra crosswalks may be with traffic control or in the middle of a block. Marked zebra crosswalks - facilities to help pedestrians to cross the street - have been used in Europe since before World War II [4]. The first zebra crosswalk ever was established in London in 1868 [5].

Motor vehicle accidents result from the interplay of the pedestrian, the vehicle driver and the environment [6]. Pedestrians involved in a motor vehicle collision are at a definite disadvantage relative to vehicle occupants because of their light and fragile bodies and low travel speeds [7].

Although much has been written about optimizing pedestrian safety, there has never been a systematic study of how injury severity and mortality are affected by the use of a zebra crosswalk - in comparison to other sections of the road [7]. It has only been demonstrated that the use of some sort of marked zebra crosswalk does reduce the overall rates of pedestrian injury [4], [6].

The aim of this retrospective study is therefore to describe the injury severity and mortality sustained by adults when crossing the road, either when using a zebra crosswalk or not.

## Materials and Methods

### Setting

Our emergency department (ED) is the largest Level I center in Switzerland, with a catchment area serving about 1.8 million people, and treats more than 35,000 cases per year. Diagnostic and therapeutic management is based on current recommendations and is at the discretion of the attending emergency physician. Suspected multiple injury is assessed and treated according to the ATLS (advanced trauma life support) guidelines.

### Data collection and retrospective survey

Our retrospective data analysis comprised adult (≥16 years) patients admitted to our ED between 1 January 2000 and 31 December 2012 in relation to a vehicle crash while crossing the street as a pedestrian, either at a zebra crosswalk or not. Patients aged 16 years or more of age are defined as adults by our hospital policy; children are treated in a different emergency department within the same hospital. Children were not included in the present study. Patients were identified using the appropriate search string “zebra crosswalk” (German: Fussgängerstreifen, Zebrastreifen) in the patient demographic field of our computerized patient database (Qualicare Office, Medical Database Software, Qualidoc AG, Bern, Switzerland). Since this medical database allows instantaneous retrieval of past diagnostic reports, discharge summaries, consultations, radiographs and other relevant medical documents, the authors were able to retrospectively analyze the type of accident, the diagnostic results, and therapeutic procedures initiated in the ED. The data was obtained prospectively by the attending physician at the time of admission and retrospectively analyzed. Moreover, police and ambulance reports were screened by hand and matched with our medical database. A zebra crosswalk was defined as a marked zebra crosswalk with or without traffic light. We did not distinguish between zebra crosswalks at an intersection or in the middle of a block. Data on speed was estimated either by the patient himself, the police or by the paramedics. The following clinical data were extracted from the medical records: admission date, manner of crossing the street (zebra crosswalk or not), type of motor vehicle, speed, count of injury, hospitalization, duration of hospitalization, intensive care unit (ICU) admissions and in-hospital mortality. Demographic data, such as gender and age, were also assessed. All medical records were reviewed by an internal specialist, a surgical specialist and a specialist in emergency medicine. Each diagnosis was coded according to the Abbreviated Injury Score (AIS) handbook (2008) and the Injury Severity Score (ISS) was calculated for each patient. According to the AIS, each injury is coded to eight different regions (head/neck, face, spine, thorax, abdomen/pelvic contents, upper extremity, lower extremity, external). Each injury is assigned an AIS severity code, ranging from 1 (minor) to 6 (maximal, unsurvivable) according to the handbook. To calculate ISS, the scores for the three most severely injured body regions are squared and summed to produce the ISS score. Severe injuries are defined as ISS >15. Large vehicles were defined as trucks and buses. Hospitalization and in-hospital mortality were extracted from our hospital's central patient registry (SAP). Traffic participants other than pedestrians (e.g. bicycle, motorbike) (n = 129), patients with incomplete records and patients with admissions not related to street crossing (n = 77) were excluded from the analysis. For patients with duplicated records (n  =  9), the second record was excluded.

### Statistical Analysis

All statistical analyses were performed with the SPSS 20.0 Statistical Analysis program (SPSS Inc; Chicago, IL). The data were summarized using descriptive statistics (means, standard deviations, percentages and Ns). The differences in patient and injury characteristics were compared between injury types using chi squared tests for categorical variables, and t tests and ANOVA for continuous variables. Survival was estimated by Kaplan-Meier analysis and between-group differences were determined by the log-rank test. Multivariable logistic regression was used to identify predictors for injury severity (ISS>15/ ISS<15), hospitalization and mortality (three different models). The predefined variables added to the model were: gender, zebra crosswalk use /non-zebra crosswalk use, vehicle type (only in the injury severity model) and severe injury. All p values were two tailed. The threshold for significance was p ≤0.05.

### Ethical Considerations

The study was approved by the Ethics Committee of the Canton of Bern, Switzerland. No individual informed consent was obtained; this was waived by the Ethics Committee. Patients records were anonymized prior to analysis.

## Results

**Figure 1:**
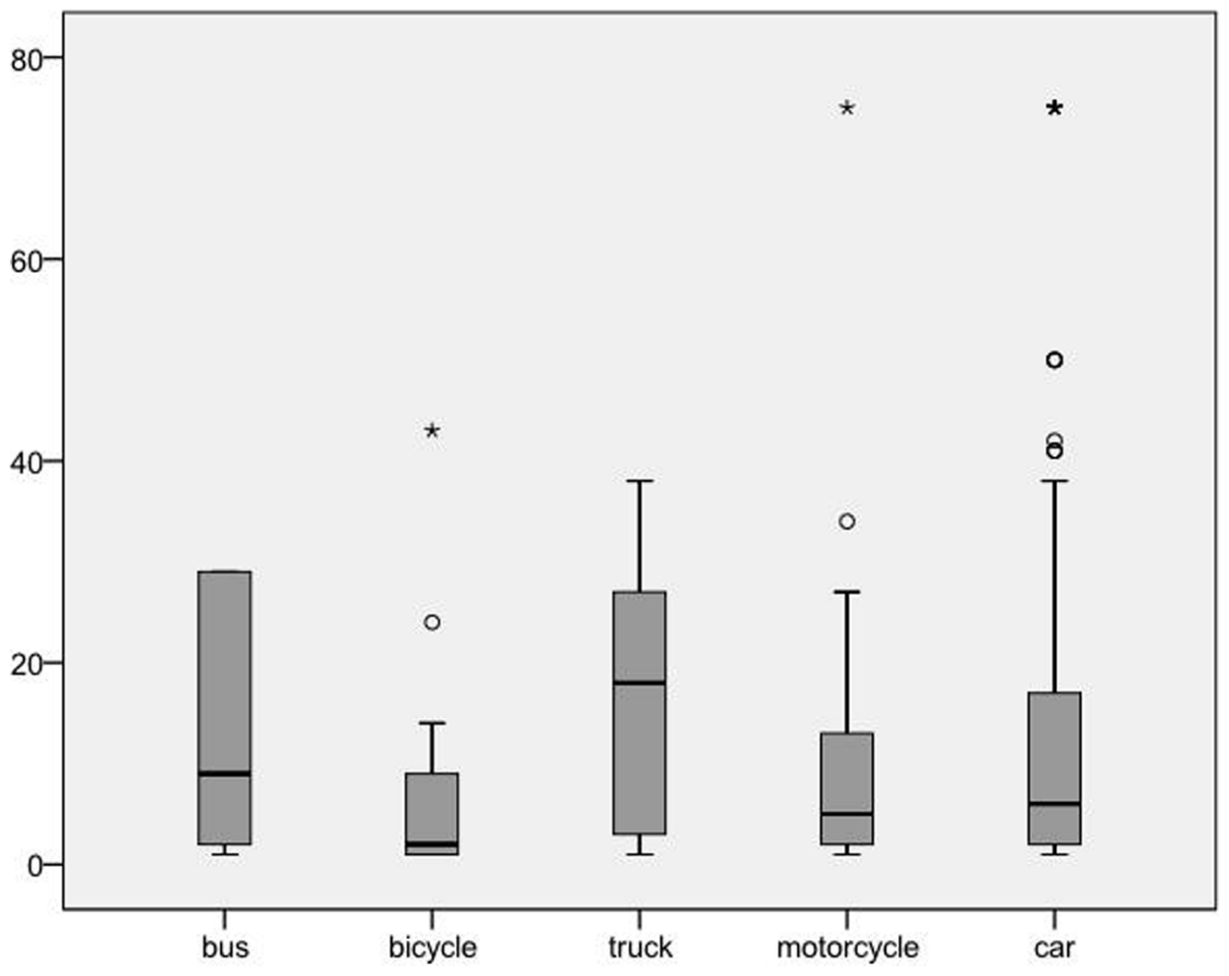
ISS-Score and vehicle type.

Of 562 patients, 347 were eligible for study inclusion. Of these, 203 (58.5%) suffered a zebra crosswalk accident and 144 (41.5%) a non-zebra crosswalk accident. For an overview of patient characteristics, see table 1. 54.2% (n = 188) of patients were female, and 159 (45.8%) male. The median age was 50.5 years (range 16–91). The mean ISS was 12.1 (SD 14.7, range 1–75). The ISS was highest in accidents involving a truck: 17.4 (SD 13.4, range 1–38; figure 1). Overall, 152 (43.8%) patients were treated as outpatients, 54 (15.6%) were admitted to the ICU (intensive care unit), 84 (24.2%) were admitted to the hospital ward, 50 (14.4%) needed emergency surgical treatment and 4 (1.2%) were transferred to another hospital. Overall 33 (9.5%) patients died, including17 (51.5%) directly in the emergency department.

**Table 1:** Patient Characteristics.

	total (N, %)	non- zebra crosswalk (N, %)	zebra crosswalk (N, %)	p value
				
**N**	347 (100)	144 (41.5)	203 (58.5)	
male/female	159 (45.8)/188 (54.2)	65/79 (45.1/54.9)	79/109	0.45
age (median, range)	50 (16–91)	50 (16–91)	50 (16–89)	0.88
				
**Motor Vehicle Type**				
car	272 (78.4)	99 (66.8)	173 (85.2)	0.0001
bicycle	30 (8.6)	24 (16.7)	6 (2.9)	0.0001
truck	15 (4.3)	6 (4.2)	9 (4.4)	0.56
bus	10 (2.9)	3 (2.1)	7 (3.4)	0.34
motorbike	20 (5.8)	12 (8.3)	8 (3.9)	0.084
				
large vehicle	25 (7.2)	9 (6.2)	16 (7.8)	0.54
				
**Mean Speed** (SD, range)	43.5 (19.2, 5–130)	47.7 (28.1, 10–130)	41.4 (5–60)	0.027
unknown speed (cases)	266 (76.6)			
				
**Mean Injury Count (SD, range)**	4.6 (3.8, 1–20)	4.7 (3.9, 1–20)	4.5 (1–20)	0.29
				
**Injury Severity (median AIS-Score, SD, range)**				
head/neck	2.4 (1.1, 1–5)	2.60 (1.2, 1–5)	2.29 (1.0, 1–5)	0.024
face	1.6 (0.6, 1–4)	1.65 (0.7, 1–4)	1.61 (0.67, 1–4)	0.74
thorax	2.4 (1.2, 1–9)	2.42 (1.1, 1–6)	2.51 (1.3, 1–9)	0.78
abdomen	2.4 (1.0, 1–5)	2.83 (1.0, 1–5)	2.27 (0.9, 1–4)	0.26
upper extremity	1.4 (0.5, 1–3)	1.39 (0.5, 1–3)	1.44 (0.5, 1–3)	0.71
lower extremity	1.8 (0.8, 1–5)	1.99 (1.0, 1–5)	1.68 (0.7, 1–4)	0.018
spine	1.8 (0.8, 1–5)	1.9 (0.5, 1–3)	1.8 (0.4, 1–3)	0.56
external	1.5 (0.7, 1–2)	1 (0, 1–1)		0.15
				
**Mean ISS (SD, range)**	12.1 (14.7, 1–75)	14.4 (16.5, 1–75)	10.5 (13.14, 1–75)	0.019
**Severe Injury**	100 (28.8)	50 (34.7)	50 (24.6)	0.041
				
**Hospitalization**				
outpatient	152 (43.8)	55 (38.2)	97 (47.8)	0.048
hospitalization	195 (56.2)	89 (61.8)	106 (52.2)	0.048
duration of hospitalization	6 (1–31)	9 (1–30)	9 (1–31)	0.57
				
**In-hospital Mortality**	33 (9.5)	18 (12.5)	15 (7.4)	0.034

**Figure 2:**
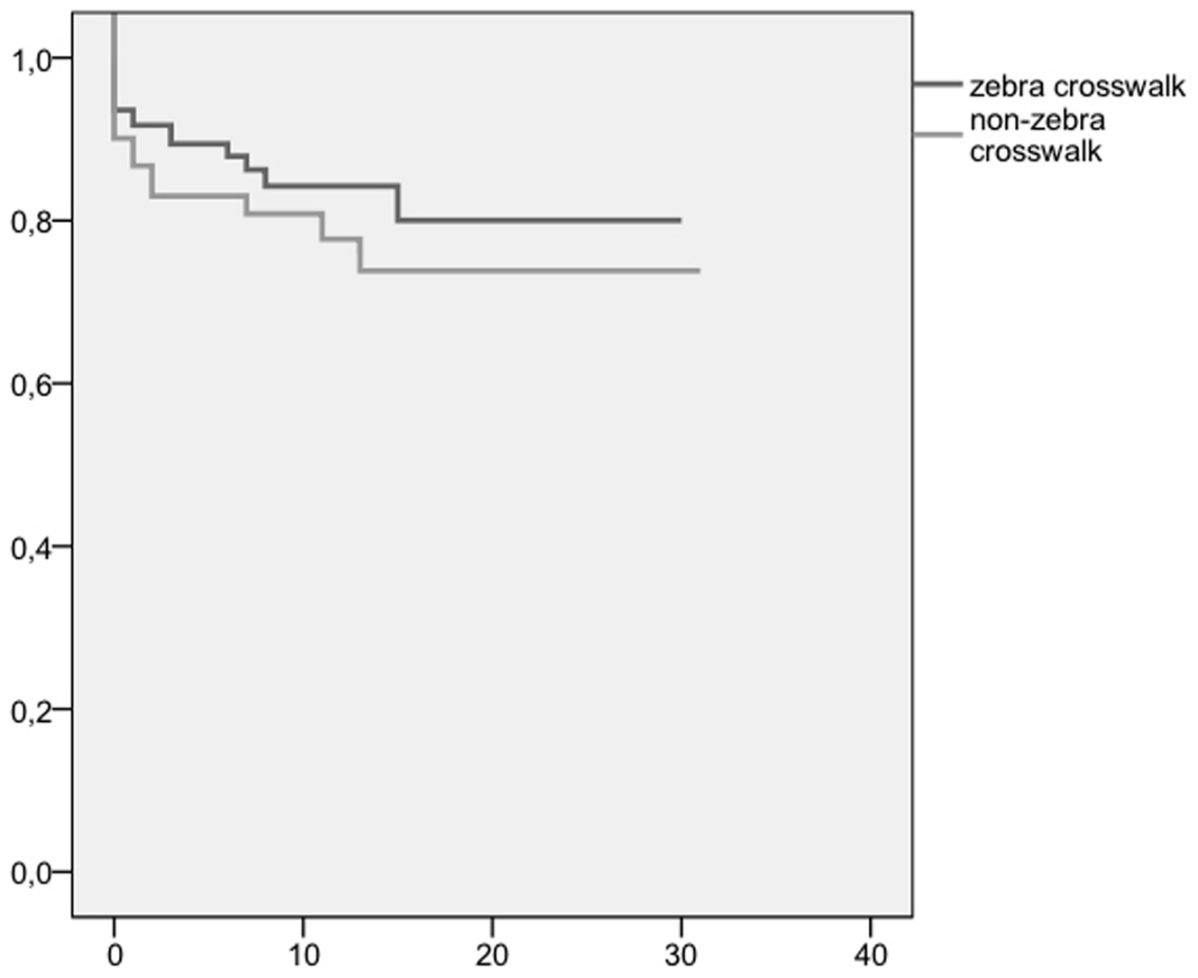
Kaplan Meier Curve for in hospital mortality (p<0.57).

For details of patients with zebra crosswalk accidents compared to non-zebra crosswalk accidents, see table 1. The two groups of patients did not differ significantly in gender or age (p<0.45, and p<0.88, respectively). Patients with a zebra crosswalk accident were significantly more often involved in accidents with a car (173 versus 99, p<0.0001), whereas patients with a non-zebra crosswalk accident were more often involved in accidents with a bicycle (24 versus 6, p <0.0001). Non-zebra crosswalk accidents were accompanied by greater speed than zebra crosswalk accidents (47.7 km/h, versus 41.4 km/h, p <0.027). The mean ISS score was higher in patients with non-zebra crosswalk accidents - 14.4 (SD 16.5, range 1–75) versus 10.5 (SD13.14, range 1–75) in patients with zebra crosswalk accidents (p<0.019). Of the patients suffering an accident involving a car or a truck, those with a non-zebra crosswalk accident had a significantly higher mean ISS than patients with a zebra crosswalk accident: 15.94 (SD 17.42) versus 10.44 (SD 13.2) for cars; 18.67 (SD11.2) versus 16.67 (SD 14.9) for trucks (p<0.002, and p<0.01, respectively). No significant difference in mean ISS score was found for other motor vehicles. Patients with non-zebra crosswalk accidents were not significantly more often involved in accidents involving large vehicles (p<0.54). Patients with non-zebra crosswalk accidents suffered more severe injuries to the head and more severe injuries to the lower extremities (p<0.024 and p<0.018, respectively) than others. Patients with non-zebra crosswalk accidents were significantly more often admitted to the hospital than patients with non-zebra crosswalk accidents: 195 (56.2%) versus 89 (61.8%) (p<0.048). Patients with non-zebra crosswalk accidents and zebra crosswalk accidents did not differ significantly with respect to duration of hospitalization or intensive care admissions (p<0.57 and p<0.64, respectively). Patients with non-zebra crosswalk accidents died significantly more often (p<0.034). The mean survival time was 2.55 days (SD 4.1, range 0–15). The mean survival time did not differ significantly between patients with zebra crosswalk accidents and non-zebra crosswalk accidents (p<0.57). For a Kaplan-Meier analysis of in-hospital mortality, see figure 2. The speed was associated with severe injury, hospitalization and mortality (p<0.0001, p<0.009 and p<0.020, respectively).

For a risk analysis, see table 2. Zebra crosswalk accidents were associated with decreased risk of severe injury (OR 0.61, 95% CI 0.38–0.98, p<0.042). Severely injured patients were significantly older than patients with less severe injuries (p<0.006). Accidents involving a truck were associated with a higher risk of severe injury (OR 3.53, 95%CI 1.21–10.26 p<0.02), whereas accidents involving a bicycle were associated with lower risk of severe injury (OR 0.16, 95%CI 0.04–0.72, p<0.017). Hospitalization was associated with advancing age (p<0.0001). Patients with non-severe injuries were at less risk of being hospitalized than others (OR 0.093, 95%CI 0.05–0.18, p<0.0001). Mortality was associated with severe injury (OR 55.03, 95%CI 12.85–235.71, p<0.0001). The type of vehicle was not associated with mortality (p<0.57).

**Table 2:** Risk analysis.

	OR	95% CI	*p value*
**Severe Injury**			
*gender*			
male	1.01	0.63–1.61	0.96
female			*Reference*
			
*zebra crosswalk*			
yes	0.61	0.38–0.98	0.042
no			*Reference*
			
*Motor vehicle type*			
bus	1.57	0.43–5.72	0.49
cycle	0.16	0.04–0.72	0.017
truck	3.53	1.21–10.26	0.02
motorbike	0.59	0.19–1.81	0.59
cycle			*Reference*
			
**Hospitalization**			
*gender*			
male	1.17	0.76–1.79	0.46
female			*Reference*
			
*zebra crosswalk*			
yes	1.48	0.95–2.28	0.077
no			*Reference*
			
*severe injury*			
no	0.093	0.05–0.18	0.0001
yes			*Reference*
			
**Mortality**			
*gender*			
male	0.64	0.30–1.36	0.25
female			*Reference*
			
*zebra crosswalk*			
yes	0.55	0.27–1.14	0.11
no			*Reference*
			
*severe injury*			
yes	55.03	12.85–235.71	0.0001
no			*Reference*

## Discussion

A total of 347 patients with road crossing accidents with known status with respect to zebra crosswalk use were available for evaluation. Almost 60% of the patients suffered a zebra crosswalk accident, whereas 40% suffered a non-zebra crosswalk accident. Patients with non-zebra crosswalk injuries were significantly more severely injured.

In our study, the proportion of patients suffering from zebra crosswalk accidents was higher than for non-zebra crosswalk injuries. It is unclear from the literature whether the risk of zebra crosswalk accidents is greater than that of non-zebra crosswalk accidents. A study by Tobey and Rouse et al found that the risk of non-zebra crosswalk accidents was up to 2.5-fold higher [8], [9], whereas Herms et al and Ekman et al found that the risk of an accident was 2-fold higher with a zebra crosswalk of any type [10], [11]. A study by Rothman et al on injury severity in accidents on zebra crosswalks with and without traffic control showed that the risk of being severely injured is 2.55-fold greater in patient using a zebra crosswalk without signals [12]. This implies that zebra crosswalks without a right of way are associated with increased risk of severe injury [12]. Generally it must be born in mind that the ratio of zebra crosswalk use to non-use is 3∶1[10]. Therefore the absolute numbers of people crossing the street at a zebra crosswalk is much higher and it is very likely that the number of traffic accidents to pedestrians on zebra crosswalks is higher than elsewhere, simply because these are more frequented. Ekman also implied that zebra crosswalks impair safety by providing a false sense of security [6], [13]. Moreover, 40% of pedestrians incorrectly believe that traffic must stop for a pedestrian who is on the curb waiting to cross a marked zebra crosswalk [14].

To the best of our knowledge, this is the first study to investigate the severity of zebra crosswalk versus non-zebra crosswalk accidents, so that we have no figures to which we could compare our findings. But several studies have found that the severity of pedestrian injury largely depends on vehicular speeds [7], [15], [16]. At a collision speed of 50 km/h, the risk of fatal injury for a pedestrian is almost eight times higher than at a speed of 30 km/h [15]. Zebra crosswalks do alert drivers to be cautious and therefore to reduce speed [15], [17] and this may explain the difference in mean injury severity and mortality between non-zebra crosswalk and zebra crosswalk accidents. Our study also shows that the risk of severe injury is significantly higher in patients with non-zebra crosswalk accidents than in patients with zebra crosswalk accidents.

In this study, injury severity is linked with the size of the motor vehicle. We showed that patients involved in an accident with a bicycle are at significantly less risk of being severely injured, whereas patients hit by a truck are at significantly greater risk of being severely injured. Other studies have found comparable results [16], [18]. According to Tefft et al, the risk of severe injury or death is higher for pedestrians struck by trucks or vans than by cars [16]. This can be explained by biomechanics [18]. Firstly, larger vehicles are heavier and have a longer breaking distance than lighter vehicles [18]. Secondly, taller vehicles hit a pedestrian above his center of gravity, so that pedestrians will not wrap around the vehicle, but will be thrown forward [18]. Thirdly, it is more probable that the pedestrian will be run over by the vehicle [18]. It is more difficult to understand why the type of vehicle was not associated with mortality in our study. It is possible that our study population was not large enough to detect this correlation, as it only included 20 large vehicles (truck, bus).

In our study, advancing age was associated with trauma severity for both zebra crosswalk and non-zebra crosswalk accidents. This has also been found by others [16], [19], [20]. This may have several reasons. Firstly, reaction time increases with age, as aging is associated with the loss of eyesight and hearing as well as poorer coordination [21]. As a consequence, older people are more susceptible to accidents when crossing the road. Secondly, older people have lower tolerance to physical trauma and sustain more severe injuries than younger persons in comparable crashes [20].

### Limitations

Our study has to be considered with some caution, as it was conducted retrospectively and the number of patients was rather small. Moreover, our conclusions on speed are less reliable, as data on speed was not available in 77% of all cases. Furthermore we have no knowledge of the motor vehicle models and types. Therefore, the size and weight of the vehicles cannot be estimated and no pattern of injury severity was detected. Further studies would be needed for this. An additional limitation to the study is the single center design and the resulting lack of external validity. As children are admitted to a specialized emergency department within the same hospital, we are not able to give any details on injury severity and mortality in zebra crosswalk and non-zebra accidents in children. Additionally we do not have any data on whether the crosswalks were with or without traffic control.

### Conclusion

In our small, single site study, we found that accidents on zebra crosswalks are more common than those not on zebra crosswalks. The severity of the injuries from non-zebra crosswalk accidents is significantly higher than in patients with zebra crosswalk injuries. Accidents involving large vehicles are associated with an increased risk for severe injury. It is still unclear whether large vehicles are more common in non-zebra crosswalk accidents. Speed also contributes to injury severity and mortality in all accidents, but further studies on this topic are needed.

Overall, further prospective studies are needed. These should involve a larger number of patients, with detailed assessment of motor vehicle types and speed.
